# Stereotactic Body Radiotherapy Immunological Planning—A Review With a Proposed Theoretical Model

**DOI:** 10.3389/fonc.2022.729250

**Published:** 2022-01-26

**Authors:** Kumara Swamy

**Affiliations:** Aster CMI, Bangalore, India

**Keywords:** SBRT, SABR, abscopal, vascular normalization, immunotherapy, antiangiogenics, immunoadjuvants

## Abstract

In the stereotactic body radiotherapy (SBRT) and immunotherapy era, we are moving toward an “immunological radiation plan”, i.e., radiation scheduling with abscopal effect as a vital endpoint as well. The literature review of part A enumerates the advantages of the intermediate dose of SBRT 6–10 Gy per fraction, appropriate use of dose painting, proper timing with immunotherapy, and the potential of immunoadjuvants to maximize cell kill in the irradiated lesions, found to have improved the abscopal effects. Part B summarizes part A, primarily the findings of animal trials, forming the basis of the tenets of the proposed model given in part C to realize the true abscopal potential of the SBRT tumor cell kill of the index lesions. Part C proposes a theoretical model highlighting tumor vasculature integrity as the central theme for converting “abscopal effect by chance” to “abscopal effect by design” using a harmonized combinatorial approach. The proposed model principally deals with the use of SBRT in strategizing increased cell kill in irradiated index tumors along with immunomodulators as a basis for improving the consistency of the abscopal effect. Included is the possible role of integrating immunotherapy just after SBRT, “cyclical” antiangiogenics, and immunoadjuvants/immune metabolites as abscopal effect enhancers of SBRT tumor cell kill. The proposed model suggests convergence research in adopting existing numerous SBRT abscopal enhancing strategies around the central point of sustained vascular integrity to develop decisive clinical trial protocols in the future.

## Introduction

Presently, several significant developments have changed the scope of radiotherapy (RT) regarding its planning and outcomes. First is the advent of stereotactic body radiotherapy (SBRT). Technological evolution in stereotactic localization of cancer and its movement has led to very high precision therapy, limiting the dose to the normal tissue. This precision has helped to deliver extreme hypofractionation to the macroscopic disease safely.

The second has been the evolution of the concept of oligometastatic disease (OMD). In stage IV, a subset of patients has limited metastases termed oligometastases. These patients are still amenable to treatment by radical approach. There has been a gradual evolution in defining patients with OMD over the years. According to ESTRO-ASTRO consensus guidelines, safely treatable 1 to 5 metastatic lesions can be considered OMD (1).

The third development is the arrival of several newer immunotherapy agents to target cancer cells and enhance the immune response against cancer, local as well distant from the irradiated tumor, at the same time. There has been a potential breakthrough with the combined use of SBRT with these immunogenic drugs.

RT has been deemed locally immune-suppressive for a long time with its cytotoxic effects on leukocytes. This immunosuppressive action is widely used in total body irradiation (TBI) as a bone marrow transplantation-conditioning regimen. However, in recent years, the RT-induced immune-stimulatory effect has been increasingly recognized, including its ability to trigger the regression of metastatic tumors (abscopal response) distant from the irradiated field. The mechanisms of the abscopal effect encompass radiation-induced normalization of the vasculature, allowing more efficient infiltration of effector T cells; IFN-γ (type II IFN) induced the upregulation of VCAM-1 and MHC-I expression leading to the generation of the tumor neoantigen. These specific neoantigens induce the maturation of DCs and migration to draining lymph nodes with the presentation of tumor antigens endogenously triggering the priming and infiltration of antigen-specific effector T cells to the tumor milieu. This local infiltration of irradiated cells is expected to enhance the cell kill leading to a new generation of neoantigens, thus leading to a cascading effect of *in-situ* vaccination. However, SBRT also increases the infiltration of immunosuppressive cells such as tumor-associated macrophages (TAMs), regulatory T cells (Treg cells), and CD11b^+^ cells. Overall, the characteristic of triggering this process of immunogenicity is a unique functional type of cell kill named immunogenic cell death (ICD) and antigen-specific, adaptive immunity by some undetermined mechanisms (2).

The process and mechanism of immune response with SBRT, immunotherapy, and abscopal effect has been dealt with exhaustively by Tharmalingam and Hoskin (3), Bernstein et al. (4), and Buchwald et al. (5). A very comprehensive review done recently by Marcus et al. covers conventional RT to high linear energy transfer radiation, RT dose scheduling for optimum immunological response, choosing an appropriate window of opportunity, and vaccination to adoptive transfer of immunity (6). The present review paper aims to consolidate complex and interdependent factors influencing SBRT abscopal effect (part A) with a proposed theoretic model into a convergent actionable strategy to have improved and dependable outcomes (part C).

Theoretically, there are four major interrelated components to maximize consistent abscopal reaction. a) There should be an effective cell kill in radiated index site (including that of resistant cells), leading to the generation of a variety of tumor-specific antigens (neoantigens), with every mutation *in vivo*, dynamically, in the entire volume of the tumor. This strategy requires optimizing SBRT dose schedules and revisiting fractionated RT principles for enhanced cell kill in the targeted lesions. b) Need to eliminate immunosuppressive cells and further improve the tumor-infiltrating lymphocytes (TILs) in the tumor microenvironment (TME) for improved local effects. The other attribute would be to overcome immune exhaustion and non-recruitment of effector CD4 and CD8 tumor-specific lymphocytes for desired abscopal effects. c) Use immunoadjuvant(s) to amplify the *in-vivo* therapeutic vaccination effect. d) An optimum combination of all these three factors in the background maintaining/enhancing the tumor vasculature integrity. Optimizing these four local factors will have a higher potential for effector abscopal reaction. The proposed theoretical model in this article primarily focuses on optimizing SBRT to improve the cell kill and enhance immunological response in the radiated site to increase the probability of systemic abscopal response.

## Part A: Review

### Radiobiology of SBRT

#### Five R’s of Radiotherapy

To counter effectively both factors of anoxia and differential responding cells, the five R’s of radiotherapy—reoxygenation, repopulation, radiosensitivity, repair, and redistribution—have been fundamental to the evolution of modern-day radiotherapy. Fractionated RT was the basic technique that brought radiation therapy out of the dark era about 100 years ago. It is, therefore, imperative to consider these four factors to use the SBRT–abscopal interaction optimally.

Based on the four interdependent factors mentioned earlier, for the proposal of the mathematical model given in part II of this article, there are two sides to the distant abscopal effect. First is the maximization of cell kill at the irradiated site to generate varied neoantigens. The second is consequent immunomodulation influenced closely by the five R’s of radiotherapy, especially reoxygenation and consequent repopulation.

##### Reoxygenation

One of the biological advantages of fractionated radiotherapy is continued improvement in the oxygenation of surviving cells after the initial fractions. One question about SBRT would be whether the reoxygenation advantage of fractionated RT and improved cell kill advantage continues to remain in the SBRT setting. In SBRT, Kim et al. elucidated that oxygen consumption would drastically diminish after a massive death of tumor cells, and thus, the surviving hypoxic cells may be reoxygenated (7). Shibamoto et al. proposed the concept of “reoxygenation utilization rate” (RUR) in SBRT (8). Two, 4, 6, 8, and 30 fractions were 50%, 75%, 83%, 87.5%, and 97%, respectively. Therefore, the authors theorized that, unlike single fractions, six to eight fractions of SBRT treatments might be sufficient to utilize the reoxygenation phenomenon (8). This advantage of fractionated SBRT may not lie with single-dose SBRT.

##### Repopulation and Radiosensitivity

Withers et al. initially described the phenomenon of clonogenic repopulation in squamous cell carcinoma of the head and neck, accelerating after a lag period of 4 weeks (±1 week) after the initiation of RT (9). Although the onset of accelerated repopulation is not explicitly known for a particular type of cancer in the temporal timeline, 1 to 2 weeks of treatment with fractionated SBRT may be advantageous in reducing the acceleration in repopulation. This phenomenon particularly holds good for rapidly proliferating cells and may improve local control (10) in aggressive disease. Depending on the stage, the delayed acceleration can happen in slow responding tumors like prostate cancer, as late as 69 days. Since relatively radioresistant cells are in the proliferation phase (with cell kill of sensitive cells), intensifying therapy with SBRT dose schedule, with planned SBRT boost at 3–4 weeks after a priming dose of fractionated RT (concomitant boost) and 10–12 weeks (delayed boost) of initial SBRT, may be critical. The basis for this proposed plan is on the literature analysis by Garau (10) about enhanced repopulation period varying from 19 to 69 days, depending on the type and stage of cancer.

##### Redistribution and Repair

The less explored biological significance of redistribution and repair for SBRT is the limitation of these two factors. However, it is logical to presume that the more partial repairs and faster redistribution of cancer cells that are likely to occur with SBRT make them more susceptible to cell kill and dysregulated repair with a higher probability of double-strand deoxyribonucleic acid (DNA) breaks. This dysregulation of repair might be much more effective with the combined effect of SBRT and chemo-immunotherapy.

#### Radiobiology of Normalization Versus Vasculature Disruption

Microvascular damage due to extensive endothelial cell kill and consequent disruption of vasculature was the initially proposed SBRT mechanism of action. Reports by Garcia-Barros et al. indicated that microvascular disorder and death of the tissue regulates tumor cell response to radiation in the clinically relevant dose range (11). Fuks and Kolesnick showed that endothelial cell kill becomes significant above a dose of 10 Gy (12) and directly affects the cancer cells (13). Additionally, the cell kill switches from DNA double-strand breaks intrinsic pathway changes over to extrinsic or membrane–stress–ceramide pathway at a high dose (14). Genetic data indicate an acute wave of ceramide-mediated endothelial cell kill, initiated by acid sphingomyelinase (ASMase), which regulates tumor stem cell response to single-dose RT of >10 Gy (15). With the present-day technology of SBRT, it is possible to deliver such vascular disruption doses of a high order, at least to resistant subvolumes, and one could expect total tumor elimination. In addition, antivascular endothelial growth factor 2 (anti-VEGFR2) induces ASMase activation and resets ceramide rheostat cell kill if high dose RT is delivered immediately (within 24 h), sensitizing the vasculature to SBRT further. In contrast, anti-VEGFR2 microvessel normalization requires at least 24 h to manifest (15). Therefore, a concurrent combination of antiangiogenics and immediate single-dose SBRT as a combined ceramide pathway vascular disruption strategy should have been a standard approach by now.

However, the work of Moding et al. is contrary to vascular disruption as an optimum strategy for the SBRT dose schedule. Moding et al. (16–18), using combinations of recombinase technology, have generated a fresh look at maximizing the endothelial cell kill mechanism of cancer control. They utilized Flp recombinase to initiate primary sarcomas and Cre recombinase to delete ataxia-telangiectasia mutated (Atm) or Bax nuclear-encoded protein selectively in the endothelial cells of mice vasculature. With this dual recombinase technology (DRT), the endothelial cells could be either sensitized or protected from the proposed membrane damage-triggered cell kill. Deleting Bax from the vasculature did not affect radiation-induced endothelial cell death or tumor response to doses of radiation commonly used in SBRT.

In contrast, deletion of Atm in endothelial cells successfully increased endothelial cell death 24 h after radiation treatment. In most of this group, the tumor recurred despite extensive radiosensitizer effect on endothelial damage after a single dose of 50 Gy (without the tumor cell sensitization), signifying that endothelial cell death just prolonged the control rate but did not contribute to sarcoma eradication. When Atm is deleted specifically within tumor cells, which substantially sensitizes them, it increases treated tumor eradication through radiation therapy. These results in a primary cancer model system suggest that the increased long-term tumor control observed with SBRT for many tumors is not due to increased endothelial cell death.

However, Moding et al. do not exclude the vasculature as a possible target for radiosensitizers used in combination with SBRT (16–18). Additionally, tumors can re-establish their vasculature from the tumor bed rim. The results show that total targeting of endothelial cells is not critical for cancer elimination.

Drawing upon these findings of DRT, it is reasonable to propose that the ideal dose would be the one that causes maximum tumor cell lysis and preserves or normalizes and enhances the tumor and surrounding normal tissue vasculature (vascular normalization). Therefore, there is a robust case for using dual recombinase technology of Moding et al. or similar differential sensitization in future preclinical animal trials to identify/escalate the optimum dose per fraction of SBRT (to eliminate the cancer cells selectively) with any of the combination therapies. Thus, genetic modulation differentially protecting the endothelial cells might permit a higher dose per fraction with better cell lysis in the irradiated tumor with expected improved abscopal effects in animal models.

#### Which Is the Best Choice? Cell Kill or Ablation/Necrosis

The traditional teaching from the onset of modern radiotherapy is that the cell kill approach is curative and necrosis of >3% is not acceptable in the curative treatment of cancer. The introduction of SBRT and the acceptance of inhomogeneous dose distribution within the tumor require an update with these basic concepts. Based on this, although SBRT is used synonymously with stereotactic ablative body radiotherapy (SABR) presently, a distinction can be made where the predominant action of the former is cell kill (with ≤10 Gy per fraction), and that of the latter is an intentional vascular disruption consequently accepting the impact of tumor necrosis (with >10 Gy per fraction). Evidence of abscopal effects with radiofrequency or microwave ablation or intratumoral alpha therapy (19–22) and SABR is against the premise of the proposed model given in part C. However, even with reported abscopal effects, these approaches could be primarily palliative for short-term control going by the works of Moding et al. (18).

### Optimizing Tumor/Stem-Cell Lysis and Immunogenicity

There are indications that sudden disintegration of a significant number of cells in SBRT (unlike conventional RT) will lead to a massive release of tumor antigens, stimulating antitumor immunity (7). A combination of therapies along with cell lysis promoting SBRT should have the ability to generate tumor-specific neoantigens to prime DCs for *in-vivo* vaccination effect. The technique of SBRT should induce maximum bystander effects with the concurrent inactivation of immunosuppressive Tregs and MDSCs. The following literature shows the way for optimization of these factors.

#### Dose per Fraction in SBRT—Single High Dose Versus Multiple Fractions

##### Local and Distant Immunological Effects

*a). Dose per Fraction >10 Gy per Fraction as Vasculature Disruptive, Immunogenic Dose.* Single fraction 20–24 Gy causes the massive release of antigens, death-associated molecular pattern (DAMP) ligands, and Toll-like receptors (TLR) and stimulates antigen-presenting cells (APC) (23). In an animal model, fractionated radiotherapy with 5 × 2 or 5 × 5 Gy combined with the immunocytokine L19–IL2 controlled all primaries and delayed the growth in distant tumors. When compared with the medium doses, a single dose of 15 Gy resulted in complete remission of 20% of the non-irradiated tumors in addition to local control in all tumors in both arms (6), indicating immediate immunogenicity is higher for disruptive doses. With the increase in tolerance of endothelial cells selectively, as described by Moding et al. (16), a higher dose per fraction may become practically applicable without vascular disruption. The resulting combination of endothelial sparing and the opportunity to deliver >10 Gy per fraction to the indexed tumor mass is expected to dramatically change the distant abscopal response as envisaged in the theoretical model proposed in part C.

*b). Dose per Fraction ≤10 Gy as Balanced Immunogenic Dose*. There are several critical advantages of choosing ≤10 Gy compared with >10 Gy dose per fraction, presently.

- High dose RT (15–20 Gy) may permanently reduce blood flow, limiting further infiltration of immune cells and aggravating the hypoxic immunosuppressive microenvironment.
- A dose of >10 Gy led to activated M2 macrophage polarization through T helper type 2 (Th2) pathway; on the other hand, 1 to 10 Gy dose per fraction reprogrammed macrophage type 2 (M2) toward an M1-like antitumor phenotype through the T helper type 1 (Th1) pathway (24). Doses of 5–10 Gy have increased nitric oxide synthetase, which repolarizes macrophages to the proimmunogenic M1 phenotype (25).
- With an anti-CD40 agonistic antibody, 6 Gy showed equal or better abscopal responses than 10 and 15 Gy.
- Dendritic cell activation: cytosolic DNA has a crucial impact on the activation of antitumor immunity by enhancing DNA sensor cyclic GMP-AMP (cGAMP) synthase (cGAS) and its downstream effector, STimulator of Interferon Genes (STING). This cascade results in interferon-beta secretion, which in turn causes dendritic cell recruitment activation, an essential element for priming CD8 T-cell antitumor immunity. The doses above 12–18 Gy per fraction cause activation of DNA exonuclease Trex1 resulting in the degradation of cytosolic DNA attenuating the immunologic response. These studies indicate that this delicate balance between cytosolic DNA and activated Trex1 is optimal at RT for 8 Gy × 3 fractions for the emergence of the abscopal effect when combined with immunotherapy (24). A clear limit that emerges for the induction of TREX1 upregulation is by the single radiation dose and not determined by the total dose delivered (26). Although *in-vitro* studies suggest that radiation compromises the stimulatory activities of DCs, *in-vivo* models demonstrate that radiation at intermediate radiation doses 5 × 8.5 Gy enhances the ability of DCs to capture tumor antigens and promotes DC migration to lymph nodes in a toll-like receptor-dependent manner. In a murine melanoma study testing intratumoral DC vaccination, 5 × 8.5 Gy enhanced the ability of DCs to capture tumor antigens without inducing enhanced DC maturation but improving cross-priming of T cells.
- In a glioma model, high-dose radiation 1 × 15 Gy induced more marked recruitment of immunosuppressive CD11b^+^ myeloid cells than lower doses of 1 × 8 Gy (25).
- In another study, 8 Gy three times enhanced the upregulation of IFN-I (26).
- In a mouse tumor model, fractionated radiotherapy and not single-dose RT induced an immune-mediated abscopal effect when combined with anti-CTLA-4 antibody (6).
- Results published by Schaue et al. about maximizing tumor immunity with fractionated radiation in the murine melanoma mouse model showed that 7.5 Gy in two fractions and 5 Gy in three fractions affected the representation of regulatory T cells (Tregs) (27).
- Therefore, the literature favors a window of 5 to 10 Gy per fraction regarding immunological response (2), and a dose per fraction of >12 Gy appears to be counterproductive.

##### Dose per Fraction Effect on Endothelial Cells and Vascular Permeability

Endothelial cell (EC) integrity is a surrogate of vascular normalization. With doses above 10 Gy per fraction, there will be an extensive endothelial damage, causing reduced vascular flow, increased interstitial pressure, vascular collapse, hypoxia, and late extensive fibrosis (25). A dose of 10 Gy in a single fraction is the threshold for induction of cell kill in ECs, and doses of 4–10 Gy per fraction may induce tumor vessel normalization, dilation, reduced leakage, and consequently increased tumor oxygenation. A single dose of 8 Gy post-RT 4 h causes minimal damage to microvessels and the ECs, with a modest <5% reduction in perfusion. In another study, with irradiation of bovine aortic ECs with a gradient dose from 5 to 15 Gy, there were two-fold increases in flattened senescent-like cells at a higher dose of 15 Gy when compared with 10 Gy. At 15 Gy, massive endothelial cell death manifested at 2–5 weeks compared with transient morphological alterations with 5 Gy (25).

##### The Importance of Extracellular Matrix and Dose per Fraction

Generally, increased tissue stiffness and tensile strength happen due to augmented collagen deposition in solid tumors. This stiffness of the extracellular matrix (ECM) interferes with the motility of antitumor T cells, antigen–antibody interaction, and delivery of immune-chemotherapeutic drugs. This feature of tumors dramatically weakens the immune surveillance and response to immunotherapy. In addition to this, 1 × 15 Gy increased collagen-I staining in xenograft tumors in a preclinical study when excised 17 days post-RT, but not with doses 2 and 5 Gy. Essentially, the master switch for the fibrotic program is TGF-ß, which stimulates collagen production and facilitates its functions. In lung tissue in a mouse study, a single dose of 12 Gy triggered TGF-β release, which peaked after 12 h but had an insignificant rise with a dose of 6 Gy (25). Therefore, given the increasing stiffness of the ECM and the disruption of the vasculature with high dose per fraction >10 Gy, it can induce a sanctuary for the persisting resistant cells by debilitating immune-surveillance and immunological interactions and hamper the subsequent delivery of drugs.

##### Dose per Fraction and Outcomes

- *Preclinical Studies.* Poleszczuk et al., with their mathematical models, show that to maximize the immune response, the dose per fraction needs to be between 10 and 13 Gy (28). In the mouse breast carcinoma model, Dewan et al. found that a dose of 20 Gy in one fraction did not significantly improve the response. The different schedules tested found that 8 Gy in three fractions was superior to 6 Gy in five fractions inducing abscopal outcome and tumor-specific T cells (29). These results suggest a specific therapeutic dose window between 6 and 10 Gy for SBRT combined with cytotoxic T-lymphocyte-associated protein (CTLA) blockade.
- *Clinical Studies.* The trial of Videtic et al. indicated that fractionated SBRT might give better clinical results. In a randomized phase II study, they compared two schedules of SBRT for medically inoperable patients with early peripheral non-small cell lung cancer. Comparing 34 Gy in a single fraction to 48 Gy in four fractions showed better 2-year overall survival (OS) and disease-free survival (DFS)—61.3% versus 77.7% and 56.4% versus 71.7%, respectively—with lower and favorable ≥ grade III toxicity for the 48-Gy arm. Although the trend of OS favored the 48-Gy arm, their study was not powered to address survival differences (30).

*Summary of Dose per Fraction*. There is literature evidence that a single fraction of 20–24 Gy vasculature disruptive doses causes the massive release of antigens with the consequent corresponding systemic immune response (23). Also, there is literature evidence that a single dose of 15 Gy induces a higher abscopal response when combined with immunomodulators (6). However, other authors have documented inadequate local and/or systemic immune response with doses per fraction of above 10–12 Gy (6, 24–27). The contradiction could be decreased local tumor cell lysis, following vascular disruption with a higher dose per fraction above 12 Gy, after an initial surge. Also, Moding et al. have shown that tumor cell killing and not endothelial disruption helps in the long-term control of the primary mouse sarcoma by SBRT (18). Within its limitation, a study by Videtic et al. showed better overall survival in 48 Gy given in four fractions when compared with 34 Gy in a single dose. In general, the literature favors a window of 5 to 10 Gy per fraction regarding immunological response (2). However, the way forward to resolve this contradiction would be first to have preclinical trials with radioprotection of endothelial cells, facilitating the use of a higher level of vascular non-disruptive doses, fulfilling both the criteria of improved cell kill and enhanced local as well as systemic immune response.

#### Optimal Sequencing of SBRT With Chemo-Immunotherapy

The optimum scheduling would be to deliver the maximum permissible dose per fraction and total dose of SBRT without vascular disruption during the window period, which would enhance the uniform delivery of immune-chemotherapy drugs within the tumor and augment the immune stimulation.

##### Evidence Against Immunotherapy Before RT

A theoretical concern is that SBRT may interfere with the immune response if immunotherapy precedes SBRT. The mechanism presumed is the obliteration of the recently infiltrated and reinvigorated T-cell response in checkpoint inhibitor immunotherapy (5). RT of 10 Gy single dose before starting immunotherapy with L19–IL2 was not beneficial in a murine F9 terato-carcinoma model, and anti-OX40 agonist antibody was optimal when given a day following radiation during the window period of amplified antigen presentation (6).

##### Evidence for Immunotherapy Concurrent or ≤7 Days of SBRT

- *Vascular Permeability*. This would be a surrogate indicator for the drug delivery efficiency to the cancer cell. In the skin of C3H-mice exposed to local irradiation 2, 15, or 50, vascular permeability peaked 24 h post-radiation, followed by a steady decline to baseline over 3–10 days. A colon adenocarcinoma xenograft study showed that 1 × 4 Gy RT increased vascular permeability 24 h post-RT, but no difference at 72 h. In another study with a radiation dose of 5 or 15 Gy to mammary adenocarcinoma xenografts, drug administration before and after RT showed 1.2- to the 3.3-fold enhancement of probe accumulation in tumors lasting the first 2 days post-RT. These results indicate that intermediate to high doses of radiation, even if not optimal to achieve tumor control, are sufficient to enhance drug delivery (25).
- *Local Immune Cell Infiltration and Outcomes*. Several published clinical studies of radioimmunotherapy combinations report abscopal effects when used concurrently or immediately afterward, depending on the type of immunotherapy. Immune infiltration started within 2–4 days after irradiation with 2 × 5 Gy in a CT26 colorectal mouse model. RT of 2 Gy × 5 fractions increased OS when used in schedules with anti-PD-L1 day 1 to 5 and not in the schedule given on day 7. The AB16 melanoma model demonstrated infiltration of CD8^+^ T cells 5 days after irradiation with 2 × 12 Gy [6]. In a mouse study, decreased PD-L1 expression and anergy of tumor-reactive T cells were reported 7 days after the last dose of RT by Dovedi et al. (31, 32). Buchwald et al. propose that anti-PD-1/L1 and RT should be concurrent (5). In the PEMBRO-RT randomized study, patients in the SBRT arm received a dose of 24 Gy in three fractions along with standard pembrolizumab within 7 days of the last dose of RT to a single site of metastatic NSCLC. In addition to the improved response rate from 20% to 50%, patients in the SBRT arm had improvement in both median progressions free survival (PFS) and overall survival (OS), although non-significant. The finding of importance was that 22% and 4% of patients with 0% PD-L1 staining (immunologically cold tumor) had a response, respectively, in the SBRT group versus the *p*embrolizumab alone arm. Despite several limitations to the study, it was a well-designed randomized clinical trial and was the first of its kind (33).

##### Evidence With Immunotherapy With or Without Chemotherapy Beyond 7 Days for SBRT

- In a preclinical study of colon cancer as a model, MHC-II-positive DC recruitment into tumors was observed only between days after the first radiation dose 5 and 10 (25).
- In the landmark PACIFIC trial, durvalumab delivered after chemo-radiotherapy led to improved survival for patients with unresectable stage III lung cancer. Initiating durvalumab within 14 days of completing fractionated RT experienced a better survival benefit than those who started on durvalumab from 14 to 42 days (33).

##### Evidence for Immunotherapy Any Time After SBRT

With 2–5 Gy, the observation of upregulation of the immunosuppressive M2 gene signature to the proimmunogenic M1 phenotype *in vitro* and *in vivo* in a few days of irradiation lasts long for several weeks [27]. In the KEYNOTE-01 clinical trial of non-small cell lung cancer, the analysis determined that the group who received immunotherapy even at a median of 9.5 months after RT had longer OS and PFS than those who did not receive RT. This study shows the lingering synergistic benefit of radiation before, although this study has limitations as a retrospective review of a single-arm trial (33). These studies indicate that immunological interaction will continue for a long time after the initial 2–7 days, even if it is not the optimal response.

##### Evidence for Multiple Combination Immunotherapies With SBRT

- RT in combination with dual immune checkpoint blockade by anti-CTLA-4 and anti-PD-L1 or anti-PD-1 resulted in the long-term survival of mice. This improved action is due to the triple action of broadening the T-cell repertoire by RT, depletion of intratumoral regulatory T cells by anti-CTLA-4, and reinvigoration of the exhausted T cells by anti-PD-L1.
- In another study, PD-L1 upregulation resulting from a concurrent blockade of TGF-β along with 6 Gy × 5 fraction radiation when nullified by anti-PD-1 delayed the tumor recurrence and extended mice survival.
- Radiation of 5 × 5 Gy combined with a bifunctional fusion protein (M7824) blocking both TGF-β and PD-L1 led to increased tumor-specific CD8 T cells, resulting in the rejection of irradiated and abscopal tumors.
- Triple therapy of local radiation to one tumor when combined with an agonistic anti-CD137 (4-IBB) and a neutralizing PD-1 antibody induced better tumor regression in mice.
- An oligonucleotide aptamer enhanced tumor response by simultaneously targeting vascular endothelial growth factor (VEGF) and 4-1BB ligand and upregulated VEGF for 12 Gy × 1 fraction (26). These studies indicate the critical place for trials of combination immunotherapies with SBRT with overall <10 Gy per fraction.

##### Synchronization of SBRT With Treg Cell Targeting

Immune tolerance associated with cancer is responsible for a poor prognosis. Increased Treg cells, a particular type of CD4^+^ T cells, play a crucial role in immune tolerance and tumor progression (2). In a mouse model, the combined RT and anti-CD25/CTLA-4 monoclonal antibody decreased Tregs, PD1^+^CD8^+^, and PD1^+^CD4^+^ T cells, resulting in the suppression of both irradiated and distal unirradiated tumor resulting in improved OS and reduced liver metastasis (34). A minimum 5 Gy is required to set in motion the inflammatory response with immunotherapy, and in a study, a 2 × 7.5-Gy schedule resulted in similar tumor growth inhibition as 15 Gy in a single dose. Additionally, lower Treg cell numbers were present in the spleens with a 2 × 7.5-Gy schedule than the single-dose arm (6), indicating a distant immunomodulatory effect.

#### Optimization of Dose Painting: Biological Target Volume

**Figure 1:**
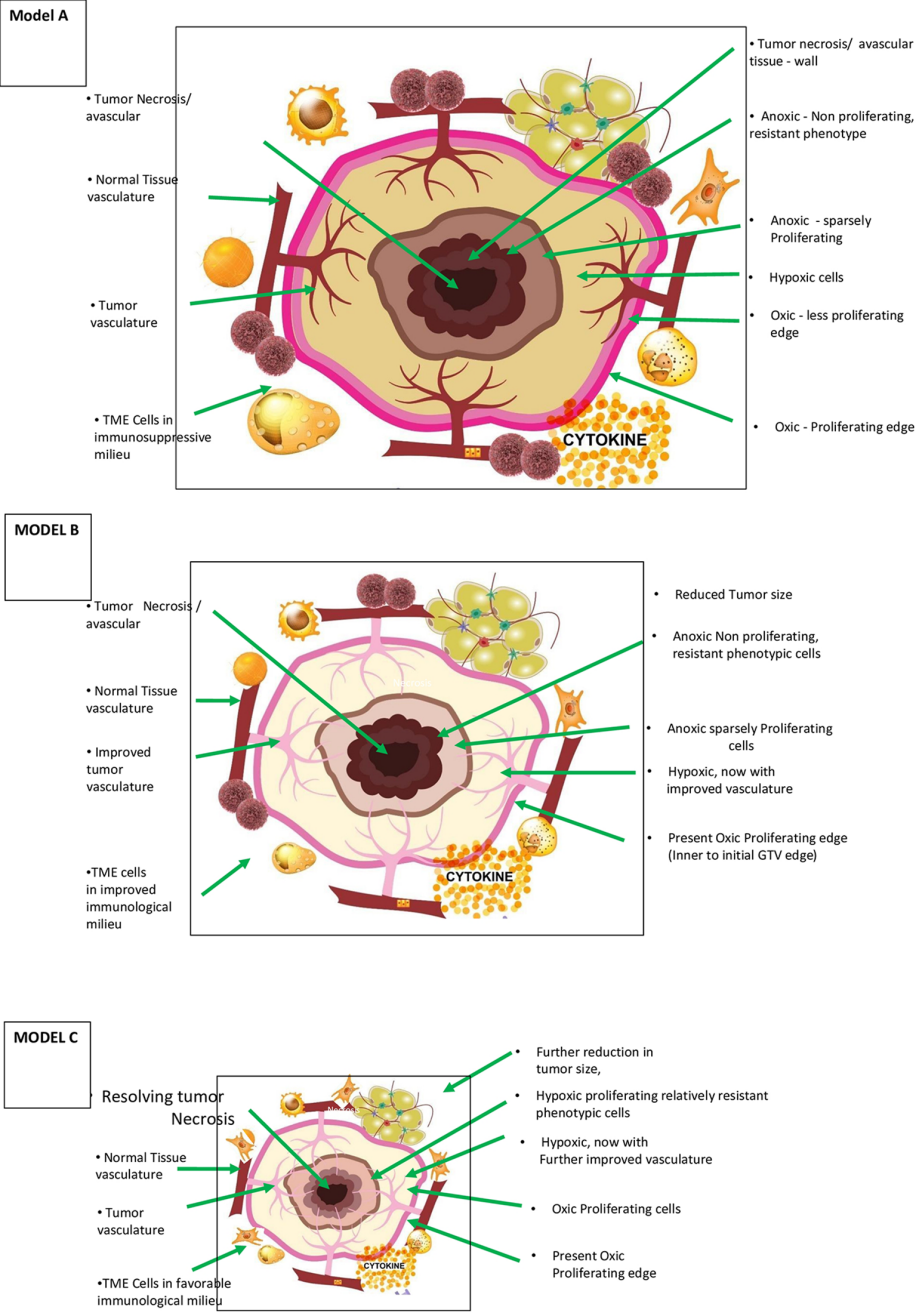
Theoretical Model A – Morphological and patho-physiological aspects of vascular-immuno-phenotypic (VIP) tumour model for SBRT and vascular sparing dose painting technique in a range of 5 – 10 Gy per fraction) (*literature evidence available – see text*) (7, 23). This is a simplified diagrammatic representation, and anoxic and necrotic volumes can be located eccentrically or irregularly distributed through the tumor mass. Varied, mildly hypoxic to anoxic cells are considered one of the major reasons for radiation/SBRT failure requiring stratagic dose scheduling and combinatorial therapies. Theoretical Model B – Morphological and patho-physiological aspects of VIP tumour model during accelerated repopulation from day 21 to 30 of start of treatment concomitant SBRT boost with or without dose painting is to be validated. Improved vasculature, decreased interstitial pressure are the other major changes during this phase. Theoretical Model C – Morphological, patho-physiological aspects of VIP Tumor model after completion of prescribed course of chemo/targeted/immuno-therapy or 3 months after radiotherapy, residual/oligo-persistance/early recurrent or oligo-progression. When present, this is the period of least tumour burden and maximum normalization with appropriate dose per fractions during initial session and when not suitable for salvage surgery, “delayed” SBRT boost with or without dose painting is to be validated.

One technique of differential dose delivery is dose painting. The cancer cells in the infiltrating edge of the gross disease are oxic and proliferating. Compared with those within, the tumor cells in the periphery are likely to be the most sensitive cells (except possibly resistant stem cells) in the entire tumor. These cells are likely to respond initially and maximally. Varied hypoxia manifests in cancer cells well within this infiltrating edge, either as a concentric gradient (**Figure 1**, model A) or eccentric/diffuse irregular fashion. Hypoxic and anoxic regions require relatively higher doses per fraction of RT for comparable cell kill. Since we have not yet found a clinically applicable effective hypoxic cell sensitizer, optimization of delivering differential doses to these varied areas by the technique of dose painting may be worth exploring diligently.

As mentioned earlier, one strategy that would be optimizing the SBRT cell kill is by differential distribution of radiation dose by the “dose-painting” technique, respecting the vascular endothelial cell tolerance. It can deliver controlled hot spots within the gross tumor volume to target the specific areas of resistant cell locations, especially cells in the “necrotic wall”. The addition of tumor perfusion image mapping can be considered to augment such approaches. The dose per fraction can be given a gradient in the hypoxic/necrotic area of the tumor from the periphery, assisted by a 3D functional imaging dose ranging from 6 to 10 Gy per fraction. Implementation of this dose gradient is easily achievable with modern-day SBRT technology. Crane et al. adopted dose-painting techniques in large hepatocellular carcinoma tumors, with photon as well as proton therapy (35). They safely used a very high dose (up to 140 Gy BED) and simultaneously integrated protector volumes, longer fractionation, and dose to the subvolume within gross tumor volume. Even though not conclusive, results were encouraging, with local control rates of 85%–90% and without significant toxicity (36).

The other technique to identify the resistant subvolumes in the SBRT targeted tumor is fluorodeoxyglucose (FDG). In a rat rhabdomyosarcoma model, by delivering a subvolume boost of 40% and a 60% dose gradient to the high FDG uptake area, Trani et al. did not find any improvement in tumor control, and in certain conditions, the tumor growth accelerated (37). This study indicates that high uptake volume may not be the critical target for subvolume boost, and studies are open for identifying PET-based SBRT biological target volume. Since PET CT scan reflects cell activity and response to SBRT, maximization of PET CT scan information for biological planning can be rewarding.

#### Improving the Tumor Vasculature

Following SBRT, the indirect effect of radiation and fixation damage by free radicals in the presence of oxygen persists for 6 to 12 weeks with continued cell death. It leads to the hypothesis that, during the post-SBRT potential lethal damage fixation period, cells would continue to be susceptible to cell kill with the local immune response and abscopal response. For this local immune response to occur, at least a skeletal tumor vasculature needs to be present post-SBRT.

The first step is maintaining the vascular integrity with an appropriate SBRT dose schedule. As discussed above, >10 Gy per fraction is likely to induce reduced perfusion, EC cell kill, increased collagen deposition, and increased hypoxia with ensuing worsening of immunosuppression. Less than 10 Gy per fraction doses promotes the dilation, normalization, vascular integrity of existing vessels, pericyte recruitment, collagen deposition unchanged, and maturation of surviving vessels (25), along with limited/recoverable damage in ECs.

##### Role of Antiangiogenics

The second step is the improvement of the vasculature with combination therapies. The exploitation of normalization action of antiangiogenics is by optimally combining with SBRT. Usually, after commencement of antiangiogenics, starting in 1–2 days, normalization of tumor vasculature results in a reduction in tumor hypoxia, a drop in interstitial tumor pressure, improved tumor perfusion, and a decrease in the peritumoral edema, and the majority of evidence comes from preclinical studies in mice subjected to continuous antiangiogenics therapy. These vascular normalization features were eventually lost and replaced by pronounced vascular regression in mice subjected to continued antiangiogenics therapy. These temporal changes demonstrated the existence of a “normalization window”. Usually, this vascular normalization “time window” persisted for at least 28 days. There was “uncoupling” of the timing of different aspects of vessel normalization (i.e., vessel size and permeability) in clinical studies, not observed in preclinical studies due to shorter observation time. Clinical MRI studies also showed changes in patients on toxicity-related “drug holidays”, and the normalization phenotype reversed while patients were off the drug. The normalization window opens in human patients with GBM as early as 24 h after cediranib therapy commences and lasts at least 28 days (38). Antiangiogenics beyond the window period carry the risk of increased tumor hypoxia, in turn aggravating immunosuppression in a dose-dependent manner (39). These findings have several implications.

Typically, antiangiogenics are administered on the same day as chemotherapy in clinical practice. A delay of chemotherapy drug delivery by a specified time after antiangiogenic administration allowing normalization to set in can enhance the response. Proper synchronization of antiangiogenics can enhance the efficacy of immunotherapy, independent of the other effects of VEGF suppression (38).

##### Other Molecules

Other than antiangiogenics, several molecules presently used in cancer therapy have the component of vascular normalization. A review article by Karar and Maity innovatively illustrates that a specific class of drugs, human immunodeficiency virus protease inhibitors (HPIs) (nelfinavir, amprenavir, and saquinavir), blocks the PI3K–Akt signaling axis. Nelfinavir decreases hypoxia-inducible factor-1α and VEGF expression *in vitro* and *in vivo* (36). Pore et al. noted that nelfinavir improves tumor oxygenation in A549 lung carcinoma xenografts (40). Qayum et al. found that nelfinavir treatment normalized the tumor vessels and observed that they were more regular with increased interbranch length and reduced tortuosity (41). Results with nelfinavir are very similar to the one with erlotinib. Erlotinib, followed by radiation, inhibited tumor regrowth to a greater degree than radiation alone (42). These reports open up new avenues in improving the tumor vasculature, possibly influencing the abscopal response, and can be used in SABR combination therapies.

#### Immune Metabolism

The other important dimension in TME is immune metabolism, which needs exploration in combination with SBRT. Activation of an interconnected complex series of processes involving inflammation, immunomodulation, revascularization, cycling hypoxia (which directly affects radiosensitivity), immune metabolites, and radiation-induced fibrosis is observed in the TME (43). The immune cells also compete with cancer cells for nutrients, essential metabolites, and oxygen (44). All immune cells need to adapt to navigate a punitive metabolic environment created by the cancer cells. Hypoxia induces the generation of metabolite adenosine that is highly suppressive of cytotoxicity by natural killer (NK) cells. mTOR is a critical driver of NK cell metabolic reprogramming (45). Therefore, exploitation of this pathway can enhance natural killer cell activity.

#### Immunoadjuvants and Abscopal Effect Enhancers

Several constituents can enhance the primary abscopal interaction between SBRT and immunotherapy agents (abscopal effect enhancers). Communicable diseases are primarily under control due to several vaccines with adjuvants contributing to their efficacy. Like conventional vaccines, if we can incorporate an effective adjuvant that can enhance this immune reaction to SBRT *in vivo*/*in situ*, it would answer the need for therapeutic cancer vaccines.

The objective of a combination of RT with different immunotherapeutic modalities is to induce action at independent levels using dendritic cells, natural killer cells, conjugated antibodies, and immune checkpoint inhibitors. Radiation of 2 × 8 Gy boosted the immunogenicity of unmethylated cytosine–guanine with oligonucleotides even in poorly immunogenic mouse breast carcinoma (6).

#### Concept of Patient-Specific Neoantigens in Modulating the SBRT

After the initial enthusiasm, SBRT has not shown abscopal effects at non-irradiated sites to the expected level compared with molecularly defined vaccines. The most important reason could be that the flooding of non-mutant peptides will dilute the neoantigens released coming in the way of organized specific mutation-oriented antigen presentation. Recent technological innovations have made it possible to dissect the immune response to patient-specific neoantigens that arise because of tumor-specific mutations. Recognition of such neoantigens is now critical (46). One could also attribute the failure to immune exhaustion and non-recruitment of effector CD4 and CD8 lymphocytes to abscopal sites. Augmentation of such specific neoantigen response or inactivation of non-mutant peptides along with SBRT would be a valuable area of trials.

Compared with traditional RT, a single dose of 20–24 Gy SABR generates more DNA double-strand breaks, minor DNA damage repair, and massive release of antigens. It releases more death-associated molecular pattern ligands and induces Toll-like receptors activating immune cell responses (23). In contrast, multiple fractions of radiation can produce an increased number and diversity of tumor neoantigens, unlike in a single fraction (33). Trials will be needed to deal with this contradiction when endothelial tolerance is no longer an issue, even with SBRT doses >12 Gy per fraction.

#### High Linear Energy Transfer SBRT

High linear energy transfer (LET) radiation is expected to have more significant immunogenic potential than photon radiotherapy due to Bragg peak effect, higher ionization density, RBE of 1.1 (proton) to 3 (carbon ion), higher unrepaired damage leading to more complex clustered DNA lesions with genomic instability ending up in micronuclei and neoantigens with greater diversity, and less irradiated leukocytes. Compared with HIF-1 stabilization, a photon radiotherapy (PRT) feature, contrarily, carbon ion radiotherapy (CIRT) attenuated HIF-1 signaling. CIRT is more effective against cancer stem cells residing in the hypoxic niche than photon radiotherapy. Largely, high LET radiation will be expected to be more effective than immunotherapies in hypoxic tumors. CIRT formed less distant metastases in the mouse osteosarcoma model than photons after exposure to an isoeffective single dose of 10 Gy (5 GyE). With greater efficacy against the primary tumor, the CIRT dose might be facilitating the development of the protective immunological memory (6).

In an osteosarcoma mouse model, CIRT alone reduced the number of lung metastases more efficiently than PRT, and in combination with IT, both radiation types suppressed metastasis outgrowth, but with greater efficiency for carbon ions. However, using the same physical dose of 10 Gy (not biological equivalent dose) in both groups might have biased the study. Results are awaited from the majority of ongoing trials (6). According to the present author, using better normal tissue sparing, higher immunogenic potential with high LET radiation appears to be encouraging; yet, preclinical studies are required to identify the optimum dose with attention to vasculature integrity. The same principles hold good for FLASH radiotherapy, an additional advantage being its ability to spare the vasculature better.

### Dose Versus Toxicities: Newer Drugs

In the PEMBRO-RT trial, in the SBRT plus immunotherapy arm, 12/35 (34%) had grade 3+ toxicities. On another phase II trial, 4/29 (14%) patients with advanced lung cancer were treated with SBRT followed by maintenance chemotherapy (grade 3+ toxicities). SBRT with immunotherapy showed grade 3+ toxicity rates of 7%–31% in any extracranial disease treatment, as shown by the review studies (33). The effective use of dose per fraction (presently <10 Gy per fraction), the appropriate total dose, and the technique of dose painting reduce the potential lethal toxicities with SBRT, keeping in mind unknown and unexpected toxicities of newer drug combinations.

## Part B. Summary of Literature Review

Moving away from anatomical and biological planning, we may be approaching an era of immunological planning in the field of radiation oncology by decoding “abscopal by chance” to “abscopal by design”, resulting in a statistically predictable and consistent effect. Primarily, the review data enumerated above are from animal experiments.

1. *SBRT dose and combination schedules:* Buchwald et al. concluded that optimal radiation dose appears to be somewhere between 8 and 10 Gy per fraction (intermediate dose) in one to three fractions (5) which may be the trade-off between maximum tumor lysis and minimal vascular disruption. Appropriate combinations with immunotherapy, immunoadjuvants, etc. are to be explored. The combination of vascular normalization and differential protection of endothelial cells has a significant potential value.
2. *Optimal sequencing of SBRT with immunotherapy*: for treatment-naive patients, initiating immunotherapy within 1 week of completing SBRT may lead to improved responses until the availability of more data represents a potential standard practice (33). Based on permeability and preclinical outcome studies enumerated above, the optimal time could be the second day after SBRT. However, one must consider the overall potential toxicity for the planned total dose.

## Part C. Proposed Theoretical Model

Deloch et al., after a comprehensive review of immune activation of radiation, concluded that for optimized treatment outcome, there is a need to go back to the bench side to get new insights.

While preclinical animal models show the advantage of SBRT over classical radiotherapy fractionation in immune activation, *in-vitro* model systems suggest otherwise (47). This discrepancy could be due to differential dynamics *in-vivo* vascular medium versus *in vitro*. Based on the data available today as itemized in the above review, this article proposes the following theoretical models and a way forward in optimizing abscopal effects equaling or exceeding the *in-vitro* model systems. The foundation for the proposed model is based on the premise that the first step in increasing the predictability of the abscopal provocation for cascading immune reaction is to improve the SBRT cell kill and invoke strategized immunomodulation of indexed lesions.

### The VIP Model and SBRT

The present author has proposed and discussed the hypothetical foundation, principles, and analysis of the vascular-immuno-phenotypic (VIP) model in general aspects of cancer therapy of locally advanced and oligometastases elsewhere. In brief, normalization of the vasculature (not the disruption) is *sine qua non* for cancer elimination in locally advanced and oligometastatic cancer. The hypothesis suggests that normalization of vasculature leads to the improved immunological milieu and reverses resistant phenotype to more susceptible ones (48). This article presents below the theoretical models for predictable SBRT abscopal effects revolving around the restoration of normal vasculature. The paper also presents a case for different combinations and newer drugs to adopt as part of the study protocol normalization of the vasculature and not disruption, in animal studies.

#### Prerequisite of Tumor Vasculature Normalization for Enhanced Abscopal Effects

Intact tumor vasculature is a prerequisite for the continued delivery of systemic drugs/pharmaceuticals during initial and maintenance therapy. Oxygenated cells respond better to SBRT, and thus, resistant phenotypes transition to sensitive types. The immunological milieu improves with enhanced immunogenic cell death (ICD). Continued generation and lymphatic drainage of tumor neoantigens facilitate improved functioning of antigen-presenting cells. The presence of intact vasculature is also crucial for the suppleness of ECM, which could be SBRT dose-dependent. Martinez-Zubiaurre et al. highlighted the immunoregulatory networks in the tumor stroma, indicating the critical nature of ECM that can facilitate an immune attack against the tumor (25). All these positive changes make the possibility of the abscopal effect more consistent.

#### Tumor Morphology and the VIP Profile at Diagnosis

**Figure 1** (model A) shows the components of the VIP model in the diagrammatic cross-section of cancer mass. The figure represents the spectrum of vascular distribution within the cancer mass from well-perfused to avascular areas, TME immunological milieu, and various cancer cells with diverse phenotypic profiles. It is essential to understand the ever-changing dynamics of these three components of the model as cancer therapy progresses to maximize the abscopal effect in SBRT successfully.

**Figure 2:**
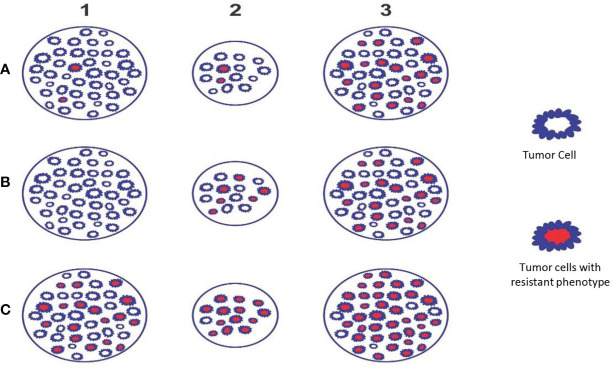
Theoretical Model D - The figure depicts the phenotypic profile and criticality of appropriate timing of SBRT. Example A: Initially few resistant phenotypes **(A1)** which survive during accelerated repopulation from day 21 to 30 of start of treatment or as residual lesion/oligo-persistence **(A2)** and these cells proliferate subsequently **(A3)**. Example B: Initially no resistant phenotypes **(B1)**; appear during accelerated repopulation from day 21 to 30 of start of treatment or as residual lesion/oligo-persistence **(B2)** and these cells proliferate subsequently **(B3)**. Example C: Initially large number of resistant phenotypes is present **(C1)**; survive during accelerated repopulation from day 21 to 30 of start of treatment or residual lesion/oligo-persistence **(C2)** and these cells proliferate to recur or metastasize **(C3)**. Situations A & B (receptor positive and differentiated tumors) indicates that SBRT can be reserved as “concomitant” or delayed boost. Situation C (e.g., receptor negative & aggressivetumors) indicates that SBRT to be considered upfront along with chemo/targeted/immuno-therapy followed by “concomitant” and/or “delayed” boosts subsequently (*preclinical trials required to establish the validity*).

Radiation-induced cancer cell kill is most effective when well-oxygenated and anoxic cells are the most resistant. The cancer tissue has nil to well-vascularized volumes, resulting in varied oxygenation, culminating in differential response to radiation. The hypoxic and necrotic area within the cancer tissue may be localized in the center, as the representative **Figure 1** (model A) depicts, or it could be eccentric or irregularly distributed within the tumor. Additionally, the cancer cells develop varied phenotypic profiles (**Figures 1**, **2**). Hence, differential targeting of the cells is required, based on this morphological and pathophysiological VIP model (**Figures 1**, **2**).

In practical terms, three strategic aspects emerge. First, with improvements in functional imaging techniques, it would be possible to have three-dimensional models of oxygenation. The importance of **Figure 1** (model A) lies in the fact that with SBRT, we have the technology to titrate the dose accordingly by creating controlled hot spots (dose painting). The dose painting has dual benefits with SBRT technology. One, a higher required gradient dose could be delivered efficiently, if needed. Two, normal tissue (including the vasculature) sparing will be more effective. Tubin et al. demonstrated the feasibility of this concept by contouring and treating only the hypoxic tumor segment. The authors delivered 10–12 Gy in one to three fractions to a hypovascularized and hypometabolic junctional zone between the central necrotic and peripheral hypervascularized–hypermetabolic tumor segment as a palliative approach and observed an abscopal effect in non-irradiated segments and nodes (49).

Second, the genomic landscape of cancer is dynamic and ever-changing in response to the fluctuating tumor microenvironment and cancer-directed treatment. This change requires an adoptive personalized approach in the SBRT delivery. The major categories of cells with different phenotypic profiles are stem cells, especially in a vascular niche or growing edge; anoxic clonogenic cells in the wall of necrotic areas; hypoxic/anoxic clonogenic cells in G0 phase of cell cycle; cells with differential SUV uptake; and cells with varying mutation burden. Improving imaging technology has made it possible for three-dimensional information acquisition facilitating matched approach and dose painting with SBRT.

#### The VIP Profile of Responding and Residual Lesions

Studying the tumor profile during treatment and residual tissue after completion of treatment is crucial. **Figure 1** (model B) enumerates the changing profile of cancer with treatment. During therapy with either fractionated RT or chemotherapy, decreased interstitial pressure happens due to tumor size reduction following initial cancer cell kill and improved vasculature. With SBRT, improved vasculature and perfusion can occur to the level of 83% by six fractions and 87.5% by eight fractions (8). TME also evolves, resulting in an improved immunological reaction. However, there could be accelerated repopulation (AR) of surviving relatively resistant cancer cells at this time.

**Figure 1** (model C) represents the tumor profile 6 weeks to 3 months after completing chemotherapy or conventional RT/SBRT. At this time, the residual disease, if present, is likely to have vascularized to the maximum possible extent due to shrinkage. Pathophysiologically, individual tumor cells are either hypoxic or oxic with nil/least number of anoxic cells possible. Theoretically, this period could be the window of least tumor burden in the timeline of cancer therapy of a particular patient. Also, if left untreated, it may not regress further. It can start repopulating with the development of resistance to the treatment, forming a potential source of recurrence and reseeding for metastases. The cells in this residual mass are most likely to be very resistant stem cells, if present, and require salvage surgery or SBRT boost in locally advanced disease in response to oligo-persistent/oligo-progression situation.

The dosage protocol should be such that it could, on the one hand, cause maximum possible tumor cell kill. On the other hand, it should retain the viability of the TME vasculature, ECM suppleness, and continued immune response. This balance is the way forward to convert “abscopal effect by chance” to “abscopal effect by design”. Hence, it is crucial to consider the interplay of the several factors enumerated above in the literature review in the background of the fundamental principles of the VIP models presented.

### VIP Models and the Proposed Innovative SBRT Schedule Harmonization

#### Possible Role of Concomitant SBRT Boost During Accelerated Repopulation at Day 21 of RT (Figure 1, Model B)

As discussed earlier, accelerated repopulation occurs around the third to fourth week of the first dose of RT. This period might be a suitable window period for SBRT to improve the cell kills in newly oxygenated hypoxic cells or evolving resistant phenotypes (**Figure 1**, model B). The study of the history of conventional RT indicates that of all the accelerated and/or hyperfractionated techniques tried, the accelerated hyperfractionated concomitant boost technique, the second fraction of the day delivered after >8 h starting from day 21, was the one that had encouraging results in the pre-cisplatin era (cf. in the concurrent technique second fraction begins from day 1). Overall, in a meta-analysis of six clinical trials, having 988 patients, Matuschek et al. concluded that accelerated RT techniques did not improve locoregional control or overall survival in high-risk patients. Additionally, acute if not late radiation toxicity was more frequent (50).

Nevertheless, when looking at the results of Ang et al., in their multi-institutional, prospective, randomized trial, the comparison between conventional radiation and concomitant boost in high-risk postoperative patients showed that the “concomitant” arm had significantly better locoregional control and overall survival without increasing the toxicities. This improvement in outcomes is after considering the postoperative period and overall radiation time together (51). Therefore, the institution of preclinical trials about the feasibility of giving SBRT boost to the gross tumor volume (GTV) anywhere between days 21 and 30 after the initial course of fractionated RT not only to increase the intensity of treatment but also to make use of the potential enhanced cell kill during accelerated proliferation and improved oxygenation phase could be worthwhile. Similarly, a planned approach of giving an SBRT boost 3–4 weeks (after the initial SBRT) to a smaller volume within the tolerability parameters of tumor vasculature and surrounding normal tissue would be another avenue for SBRT optimization.

#### Possible Role of Delayed SBRT Boost With Chemo-Immunotherapy (Figure 1, Model C)

Paik et al. published the results of 23 patients with 29 oligometastases treated with a split course technique (52). They delivered one to three sessions of SBRT course initially and a second course at around 4 weeks with a range of 18–60 days, to reduce the dose to critical organs based on the observation of faster rates of tumor regression with SBRT compared with that of conventional RT. Their data showed a partial response in 55% of the patients before the second course of SBRT (52). The triple therapy of anti-PD-1, a checkpoint inhibitor, and indoximod, an immune-metabolic adjuvant, together with 2 × 12 Gy RT induced a rapid tumor regression in mice bearing melanoma. In this trial, eventual tumor recurrence was associated with increased cell kill of intratumoral T cells. Re-irradiation with 2 × 10 Gy at a late tumor regression phase or after relapse cured the majority, which correlated with more memory T cells in the tumor-draining lymph nodes and spleen. Also, re-irradiation effectively delayed the relapse in mice having poorly immunogenic mammary carcinoma (6). This finding could signify that the delayed boost schedule fits in with synchronizing the SBRT boost during the phase of least tumor burden and possibly having the most resistant residual cells. In addition, treating selected patients with residual lesions with reduced irradiation volume would be a plus point in reducing the overall side effects and is worth exploring.

### The VIP Model and Ablative Approaches

Abscopal events do happen with intense ablative approaches. Presently, results like these are available primarily in recurrent cases and patients who undergo multiple therapies sequentially. Compiling observational study outcomes in these recurrent patients who undergo a combination of SBRT/SABR, radiofrequency ablation/microwave ablation (RFA/MWA), or alpha therapy in an opportunistic sequence with immunotherapy would help generate hypotheses for the optimization of SBRT abscopal ramifications with ablative procedures. Nonetheless, according to the components of the VIP model, these approaches, even with abscopal effect, at best could have prolonged palliative benefit in locally advanced/oligometastatic (>3 cm) malignancies. Ablative therapies like RFA or MWA require at least 2 cm of normal tissue around the tumor. Else, there were increased chances of local recurrence (53). Therefore, preclinical trials are required to evaluate the cure rate rather than the local control rate with ablative versus multiple factions incorporating the DRT or similar differential sensitization principles. However, in animal trials, one caveat is that proper evaluation of long-term survival may not be possible with the limited life span of mice.

### The VIP Model and Convergent Research

- Worldwide cancer research is happening by diverse groups with varied targets. It is like cutting “branches of a tree”, which can regroup at the same place or elsewhere through innumerable “branching pathways”. Since there are enough data, primarily through preclinical animal studies, the planned gamut of research can also incorporate the fundamental “root” factor, thus facilitating the convergence of different strategies to one point. For this, the primary factor to be considered is having in any study a trial arm monitoring preservation, or enhancement of tumor vasculature integrity, since intact vasculature could be a prerequisite for cancer elimination, especially in the locally advanced and oligometastatic setting. This approach will target the specific phenotypes by targeted therapies and tackle the “source code” as well. Otherwise, we might miss the usefulness of a new drug that would not reach the target due to poor uneven distribution within the tumor tissue in a trial.
- Future trials should incorporate DRT technology enunciated by Moding et al. (18) or similar differential sensitization to increase the cancer cell kill, simultaneously protecting the endothelial cells to rework the optimum dose of ICD.
- The other potential approaches are as follows: a) cyclical SBRT of <10 Gy per fraction before each dose/cycle of immunotherapy; b) cyclical administration of antiangiogenics making use of the window of normalization “off and on” with the delivery of SBRT with each normalization “on” after planned antiangiogenic “drug holidays”; c) enhancing the vasculature during cancer-directed therapy; and d) immunoadjuvants for *in-vivo* vaccination effect and immune metabolites as abscopal effect enhancers.

### Proposed Strategy

After the initial course of SBRT, 8–10 Gy per fraction concomitant boost dose SBRT at 3–4 weeks, and a delayed SBRT boost dose of 8–10 Gy per fraction at 6–12 weeks with “shrinking volume,” the dose-painting technique is worth exploring. Studies are required to identify the optimum immunogenic dose between 6 and 10 Gy. The total dose of initial, concomitant, and delayed boost put together depends on the size of the lesion (treatment volume), surrounding critical structures (organs at risk), and response, respecting the permissible dose constraints. This strategy appears to satisfy the requirements of the theoretical VIP model, e.g., integrity of the vasculature, handling of accelerated repopulation at 3–4 weeks, and residual resistant phenotypic stem cells at 6–12 weeks, resulting in the maximum generation of varied evolving immunogenic tumor antigens (**Figures 1**, **2**). Additionally, after consolidating all the above reviews, the author proposes four other supplementary requirements along with the fundamental requirement of vascular and endothelial cell preservation to optimize SBRT (**Table 1**). For combinatory treatments of RT and IT, drugs aiming for normalization of vasculature should be prioritized along with drugs aiming to reduce potential RT-mediated immunosuppressive effects since the normalization of vasculature is required for overcoming the local immunosuppressive milieu as well as to facilitate the distribution of immune-stimulatory drugs within the target.

**Table 1:** Proposed fundamental and supporting requirements to facilitate augmented abscopal effect.

Requirements and Strategies	Effects
1. Fundamental prerequisite: minimal disruption of tumor and normal tissue vasculature	Enhances oxygenation, fixes potentially lethal damage, and maintains sensitivity to further doses of SBRT; enhances tumor hostile TME, e.g., normal physio-biochemical response and immune metabolism; permits continued delivery of subsequent doses of drugs; encourages cancer cell–TILs and NK cell interaction; carries tumor neoantigens and primes cancer killer cells for abscopal action, reduces side effects
2. Harmonization of a combination of therapies with SBRT/radiosensitization of cancer cells	Additive/synergistic (rarely antagonistic) effects; augments immune stimulation, handles heterogeneous cancer cell population
3. Enhancing tumor vasculature (under cover of anticancer treatment) or increasing resistance of endothelial cells or both	Converts hypoxic and anoxic cells to oxic cells to sensitize them for subsequent doses of SBRT, clears degraded and dead necrotic cell products, continues to present neoantigens, avoids endothelial senescence and long-term toxicities
4. Immunoadjuvants and abscopal effect enhancers	Has multiplier effects of abscopal reaction, facilitates *in-vivo*/*in-situ* therapeutic vaccine induction
5. Immunological RT planning: appropriate dose per fraction, dose painting, and concomitant SBRT boost	Optimizes SBRT for abscopal effect, improves cancer stem cell kill, improves immunological milieu, maintains supple ECM, and simultaneously reduces the side effects
6. SBRT as delayed boost	Targeting residual resistant population and stem cells to prevent recurrence and reseeding; reduced side effects

**Table 2** shows the proposed optimum utilization of the VIP model for the SBRT harmonized combinatorial schedule. This approach also fulfills the requirement of delivering the maximum possible tolerable dose with acceptable side effects. The method is similar to the well-established medical oncology practices using proper dose per cycle, total dose, distributed over an optimum period depending on the type of cancer.

**Table 2:** Proposed SBRT dose schedule approaches with or without harmonized combination therapies matching the tumor profile of the vascular-immuno-phenotypic (VIP) model.

SBRT Dose Schedule/Strategy	Tumor Profile Targeting *vis-a-vis* Normal Tissue Effects
Single or high dose multiple (>12 Gy per fraction)	Vascular disruption with subsequently increased hypoxia; even higher dose may not be adequate to kill resistant cells; a one-time flood of antigen generation and presentation; dose modification not possible for concurrent side effects; DRT or similar differential sensitization of cancer cells sparing the ECs—preclinical trials required
Intermediate dose, multiple (<10 Gy/fraction)	One of the fundamental five R’s of RT “reoxygenation” is accounted for to an extent resulting in increased levels of hypoxic cells lysis; vascular and ECM integrity maintained; multiple time, scalable neoantigenic presentation; leeway for optimization of total tolerable dose of SBRT based on concurrent side effects; DRT or similar differential sensitization; preclinical trials required to identify optimum dose between 6 and 10 Gy.
Intermediate dose, multiple (<10 Gy/fraction), boost—concomitant or delayed	Targets proliferating resistant cells and stem cells; vascular and ECM integrity maintained with better oxygenation and drug delivery; continued scalable neoantigen presentation; optimization of volume and total tolerable dose of SBRT based on response with limitable “titratable” acute side effects; preclinical trials required.
Cyclical” SBRT <10 Gy/fraction, multiple fractions before each immunotherapy dose and/or cyclical antiangiogenics	SBRT as sensitizer secondary to primary therapy, i.e., chemo-immunotherapy; optimum reoxygenation; repeated scalable neoantigenic presentation; vascular and ECM integrity maintained; optimization of total tolerable dose of SBRT with limitable and titratable acute side effects; preclinical trials required.

The earlier preliminary version of this article is available in preprints (54).

The limitation of this article is the lack of direct experimental evidence, which is primarily a theoretical model for preclinical trials. The reference article of the author quoted is also a hypothesis article. The literature review in the first part, which is the basis for this model, has the significant limitation of not many closely relevant clinical studies available to be included. The model has focused on different cell types rather than on tumor types.

The strength of the article is a comprehensive distinctive compilation of available literature supporting the theoretical model.

## Conclusions

SBRT can be a powerful immunological weapon by amplifying indexed tumor cell kill with a strategy of harmonized combination therapies. The foundation for the proposed model is based on the premise that the first step in increasing the predictability of the abscopal provocation for cascading immune reaction is to improve the SBRT cell kill and invoke strategized immunomodulation of indexed lesions. This improved cell kill in irradiated lesions requires a proper dose schedule, immaculate use of the window of opportunity, deactivating immunosuppressive factors in TME, and successfully generating tumor-specific neoantigens to induce *in-situ*/*in-vivo* therapeutic vaccination matching the changing milieu. According to the proposed model, these SBRT-adopted local effects are projected to have a cascading impact on unirradiated cells as an amplified abscopal phenomenon. Clinical trials to identify an effective new therapy are time- and resource-intensive projects. There is a basic need for diverse SBRT abscopal animal trials to have a vascular normalization arm as a primary requirement to facilitate the proper distribution of the therapeutics to be tested within the tumor mass and augment the tumor cell kill. This singular strategy is likely to be a practical convergent point for decisive clinical trials in the future.

## Data Availability Statement

The original contributions presented in the study are included in the article/supplementary material. Further inquiries can be directed to the corresponding author.

## Author Contributions

The author confirms being the sole contributor of this work and has approved it for publication.

## Conflict of Interest

The author declares that the research was conducted in the absence of any commercial or financial relationships that could be construed as a potential conflict of interest.

## Publisher’s Note

All claims expressed in this article are solely those of the authors and do not necessarily represent those of their affiliated organizations, or those of the publisher, the editors and the reviewers. Any product that may be evaluated in this article, or claim that may be made by its manufacturer, is not guaranteed or endorsed by the publisher.
